# Total HIV DNA: a global marker of HIV persistence

**DOI:** 10.1186/s12977-018-0412-7

**Published:** 2018-04-03

**Authors:** Christine Rouzioux, Véronique Avettand-Fenoël

**Affiliations:** 1 Laboratoire de Virologie, APHP Hôpital Necker Enfants Malades, Paris, France; 2 EA 7327, Université Paris Descartes, Sorbonne Paris-Cité, Paris, France

**Keywords:** Total HIV DNA, Real time PCR, Reservoirs, CD4 + T cell subsets

## Abstract

Among the different markers of HIV persistence in infected cells, total HIV DNA is to date the most widely used. It allows an overall quantification of all viral forms of HIV DNA in infected cells, each playing a different role in HIV replication and pathophysiology. The real-time PCR technology is to date, a precise, sensitive and reproducible technology that allows the description of the distribution of HIV infected cells in blood and tissues. The objective of this review is to present some examples which show the interest to quantify total HIV DNA levels. This marker brought an undeniable and considerable contribution to reservoir studies. Many results, both in clinical and basic research, allowed to get a large overview of the distribution of infected cells in the body, at all stages of HIV disease and during therapy. Future clinical studies aiming at reducing HIV reservoirs will benefit from HIV DNA quantification in blood and tissues, in association with other markers of HIV reservoir activity.

## Background

Among the different markers of HIV persistence in infected cells, total HIV-1 DNA is to date the most widely used marker. This marker is often considered as imperfect. However, it is the one that has brought and will bring the most results in HIV reservoir studies. The major criticism is that this marker makes it possible to quantify all forms of HIV-DNA, without differentiating the defective forms from the latent ones that can produce infectious viruses. This drawback can also be considered an advantage because it therefore allows an overall quantification of all viral forms of HIV DNA, each playing a different role in HIV replication and pathophysiology. In fact, defective proviruses participate in HIV pathogenesis, as they can produce viral antigens, incomplete viruses, can induce activation/inflammation in infected tissues, thereby maintaining viral replication and facilitating the persistence of HIV reservoirs throughout the body [1]. Clearly, all reservoir cells represent the engine of the viral infection and merit to be measured. The pathophysiology of HIV-1 reservoirs is complex and different in infected compartments and tissues, that justifies to explore multiple markers together, each one having a different meaning. The question is not what is the best marker, but rather what is the best association of reservoir markers for each program [2]. Among all reservoir markers, total HIV DNA represents one of the master pieces to build the puzzle.

## Technical aspects of total HIV DNA quantification

Total HIV DNA, also called cell-associated HIV DNA (CA-HIV DNA), is a marker of HIV reservoirs that permits the quantification of all forms of HIV DNA including stable integrated proviruses and unintegrated forms, including extrachromosomal 2-LTR, 1-LTR forms and linear forms. All these forms co-exist in infected cells during viral replication and their levels may vary among patients, according to the stages of HIV disease [3].

The most frequently used method for measuring total HIV DNA is the quantitative real-time PCR based assay [4]. HIV-1 DNA PCR assays are performed on total DNA extracts from cells. Amplification has to be done with primers and probe, targeting conserved regions of the viral genome. LTR, *pol* and *gag* genes are the most often selected, but the high viral genetic diversity has to be taken into account, especially in countries where there are high numbers of various CRF and non-B subtypes. The quantification of the copy number is based on a standard curve prepared by serial dilutions of a standard, such as 8E5 cell line containing one genome per cell. The initial quantification of total DNA by the measurement of the optical density at 260 nm (OD_260_), or by quantifying a cellular gene in parallel by PCR (such as CCR5 or albumin), is necessary to assess the cell number tested in a PCR and to calculate the frequency of infected cells per one million cells [5]. It is generally assumed that there is one copy per infected cell, in particular in latently infected cells which are dominant, especially among patients receiving a prolonged and effective antiretroviral treatment. The frequency of infected cells being very low, the objective is to quantify a rare event. Such quantification follows the Poisson probability distribution. So, whatever the technique used, it is necessary to test high numbers of cells, in order to reach low detection levels. Since the amount of total DNA is limited per PCR well, it is often necessary to test several replicates, in order to increase the number of cells tested and to estimate the frequency of infected cells, as well as possible (especially in case of very low frequency). The Boston patients and the Mississippi baby cases confirmed that the latent reservoir can persist at a level below the limit of detection of current assays, allowing the rebound of HIV infection months later. An ultra-sensitive protocol could be used by testing six to eight replicates, to explore a high number of cells and detect low levels as it has been done for Elite controllers and Post Treatment Controllers (also called VISCONTI patients) [6–8]. The same technology has been also developed for HIV-2 infected patients, having usually low reservoir levels. A new assay for HIV-2 DNA quantification based on the same technology has also been developed [9].

The quantitative real-time PCR offers a number of technical advantages, making the total HIV DNA the most widely used marker for exploring HIV reservoirs. There are multiple reasons for this situation: the assay is the most feasible and reproducible, it is quick, easy to perform, precise, accurate, sensitive and with a large dynamic range of quantification. Compared to other assays, such as QVOA which may need more than 100 ml of fresh blood, small amounts of blood or tissue can be tested and samples can be stored frozen before testing. Moreover, it is less expensive and time consuming. The technique has a good reproducibility, as shown with the intra-laboratory control reported in a recent review [10], the Inter-assay coefficient of variation was at 0.07, in the same range than a recent one at 0.15 [11]. Lastly, the results obtained, within inter-laboratories control, has confirmed that this technique could be implemented within multi-centric protocols and clinical trials [12].

One standardized quantitative assay based on real-time PCR has been commercialized (Biocentric, Bandol, France). This has enabled access to both basic research and clinical research teams to use the same quantitative tool, making possible comparisons between studies [13].

Similarly to assays which have been developed for HIV RNA quantification, the total HIV DNA real-time PCR assay is easy to be adapted to automated nucleic acid extractors and real time-PCR machines. It takes around 4 h to test more than 80 samples within one run, making this technique well adapted to test large series of samples. This has provided good statistical power, which was very helpful to demonstrate that the total HIV DNA level in Peripheral Blood Mononuclear Cells (PBMC) predicts disease progression and to explore the dynamics of this marker, using mathematical models [14, 15].

Some teams also propose to use the digital droplet PCR (ddPCR) technology, which can precisely quantify with accuracy and reproducibility nucleic acids such as total HIV DNA [16, 17]. Jones et al. demonstrated that a six logs linear dynamic range of ddPCR is approaching the seven logs, achievable by real-time PCR. Its drawbacks include the cost of equipment and the complexity of the assay, while real-time PCR is widely used in research and clinical laboratories [18]. This technique has been mainly applied to blood samples, and results obtained are very close to those obtained with real-time PCR [19]. On the whole, ddPCR gives accurate quantification of low levels of HIV DNA, but false positive results with ddPCR may occur [17, 19, 20]. Less frequently, other technologies have been proposed to quantify total HIV-DNA, such as seminested real-time PCR, nested PCR assays [21–24]. Furthermore, PCR with amplification of extracts, at limiting dilution, and detection by real-time fluorescence confirmed by melt curve, is also commonly used [25].

Lastly, in order to explore the consistency between blood reservoir markers, a comparative analysis of measures of markers, in acute and chronic patients, reported correlations between markers. This includes correlations between total HIV DNA and integrated HIV DNA and HIV DNA in rectal CD4 + T cells [26]. Of course, the correlations cannot be perfect, each marker having a different meaning and playing a different role in HIV reservoir persistence.

## Total HIV DNA quantification applied to many kinds of samples

The quantification of total HIV DNA permits to estimate of the total number of all infected cells, resting or activated, present in blood, in tissues and biopsies.

### Quantification in peripheral blood

Total HIV DNA level is mainly quantified within pellets of PBMC, usually separated from plasma on Ficoll–Hypaque density gradient. The technique can also be readily adapted to quantify total HIV DNA directly on whole blood samples, the frequency of infected cells is then obtained by taking into account the blood formula. The predictive value of total HIV DNA has been reported, whatever the mode of expression of the results, per million CD4 + T cells, per million PBMC, or per ml of whole blood [27].

Because blood is the most accessible and easily quantifiable compartment, the majority of other reservoir markers, whether exploring the number of producing cells or measuring transcriptional activity, are also done mostly from the peripheral blood. So, the majority of studies have explored blood reservoirs, despite criticism that the majority of infected cells are in tissues and that the blood contains only a small number of infected cells. It would therefore be unrepresentative. It is true that the normal distribution of lymphocytes is 2% in the blood, while it is 98% in tissues. However, such a criticism does not seem to take into account the fact that the same criticism could apply to the quantification of blood CD4 + T lymphocytes and HIV RNA in plasma, whereas they are routinely used and they represent clinical markers definitely considered essential. The peripheral blood reservoir quantification is the most logical and clinically feasible approach, as are the use of CD4 + T cell count and plasma HIV RNA level for patient monitoring. In addition, the total level of HIV DNA in PBMC is the only marker of HIV reservoirs for which the predictive value of the risk of progression to AIDS and death has been well demonstrated [14, 28]. This confirms that the level of HIV DNA in peripheral blood level is representative of the total reservoir. However, it is true that infected blood cells may not adequately reflect all critical events occurring outside the bloodstream, neither the whole story of HIV reservoirs.

### Quantification from sorted cells

This assay has also been developed and largely applied to the quantification of infected cells within fractions of sorted blood or tissue cells, such as CD4 + T lymphocytes, CD4 + T cell subsets, including naïve (TN) central memory (TCM), transitional memory (TTM) and effector memory (TEM). Resting T and activated T cells are also informative to the pathophysiology of HIV infection. It is also interesting to combine assays, such as total HIV DNA quantification and capacity to produce HIV RNA in cell culture, using cell sorter such as FacsAria on the same living fractions [7, 29].

### Quantification in tissue biopsies

Total HIV DNA has proved very useful to describe the distribution of infected cells in tissues and anatomic reservoirs. That also allowed to document infected areas in which the distribution of medicinal products must be particularly important [30], including anatomic compartments, such as CNS that may also act as sanctuaries. The assay could be performed on small fragments, using a specific method for nucleic acid extraction [31]. Studies performed in non-human primate models have also benefitted from this marker with extensive quantifications in many tissues. This includes autopsies, showing the high number of infected cells, and more specifically the major role of lymph-nodes at the origin of viral dissemination throughout the organism [32, 33].

## Total HIV DNA at different stages of HIV disease

The spectrum of total HIV DNA levels in PBMC during HIV infection have been presented in a recent and extensive review, confirming the major impact of this marker to get a global overview of HIV reservoir levels in different groups of infected patients. Of note, we used the same assay in all studies, that permits to compare patient groups [10].

### During the natural history of HIV infection

The data showed that the reservoir is seeded very early in infection, with high levels in primary infection at the peak, then the HIV DNA set point is rapidly established [34, 35]. Total HIV DNA in blood and gut levels are significantly lower in patients with primary infection, at stage Fiebig I, versus Fiebig II–IV [23]. Total HIV DNA level in PBMC were also described in children [36], at the AIDS stage [37] and was found strongly associated with HIV-associated neurocognitive disorders, independent of plasma HIV RNA, indicating the neurologic impact of a larger reservoir [38, 39]. Elite controllers are characterized by a very low reservoir level, especially those bearing HLA protective alleles [8, 40].

Interestingly, total HIV DNA levels in PBMC correlate positively with plasma HIV RNA and negatively with CD4 + T cell count [14]. There is a link between HIV reservoir levels and activation [41, 42]. Lastly, several reports confirmed the high predictive value of the total HIV-DNA level in PBMC during natural history [14, 28, 43–45]. HIV reservoirs play a major role within lymph-nodes, including in the B cell follicle sanctuary [32, 33]. The distributions of HIV DNA and HIV RNA differ between gut and blood [46], and in gut associated lymphoid tissue of controllers and non-controllers [47]. Quantification of HIV DNA in kidney, as well as in adipose tissue, indicates that they can be considered as anatomical reservoirs [48, 49]. Measuring HIV DNA in genital compartments may indicate the presence of infected cells and may help to explore this risk of sexual transmission [50, 51].

### Under antiretroviral therapy

The impact of antiretroviral therapy on HIV DNA is less than that on plasma HIV RNA. The decay of the total HIV DNA in blood is faster in acute infected patients receiving early treatment, compared to a slow decrease in patients treated at the chronic stage, in whom the kinetics shows a first phase of decay in the first year, then followed by a sort of plateau [52]. On the contrary, among acutely infected patients, the decrease continues beyond 4 years of primary infection treatment, while no further decay is noted in chronic treated patients [15, 35]. The earlier the treatment is initiated, the more prominent is the total HIV DNA decrease [34]. Studies of the total HIV DNA decay dynamics in blood, during more than a decade of suppressive antiretroviral therapy, indicates a slow decline during these last years, with a remarkable stability of a plateau, balanced by homeostatic proliferation [52–55]. High total HIV DNA levels in PBMC are informative when measured at treatment interruption, as they predict a shorter time to treatment resumption, independently of the CD4 nadir [56], while low levels predict a higher probability of maintaining viral control [57, 58].

This marker has proved to be particularly useful and has shown interest in immuno- pathophysiological studies. First, the results showed that HIV DNA level in PBMC is predominantly composed of T CD4 + Central Memory Cells (TCM) in patients at the chronic stage [59]. These TCM are preserved from infection by early treatment initiated in primary infection [29, 60], while HIV DNA subspecies persist in both activated and resting memory CD4 + T cells during therapy [61]. Early antiretroviral treatment maintains the distribution with protection of the TCM [62, 63]. So, the measurement of total HIV DNA levels in PBMC contributed to show that early treatment initiation remains, so far, the best way to limit the size of the reservoirs.

Interestingly, the distribution pattern in CD4 + T cell subsets of VISCONTI patients seems to have also been frozen by early treatment, with TCM that contributes minimally to the total blood reservoir. Moreover, HIV DNA levels decrease over time in some of them: all this suggesting that the protection of TCM compartment might be necessary, and/or participates to the control of HIV replication [5, 7].

Interestingly, this marker was the only positive marker of HIV infection in the first VISCONTI child, who still presents a long-term remission with a sustained control of HIV replication since more than 12 years [64]. This marker permitted to estimate the impact of cytoreductive chemotherapy on HIV reservoir persistence [65], and the long-term impact in children and adolescents receiving treatment [66, 67]. The impact of treatments in different anatomical compartments, such as genital tract, to explore the residual risk of sexual transmission is important in the context of various levels of drug diffusion in tissues. Blips of viral replication in semen correlate with the level in PBMC, among men having sex with men on successful antiretroviral regimen [50, 68]. Levels of HIV reservoirs have been also estimated in patients receiving suppressive antiretroviral therapy, showing a true impact on different tissues and compartments, such as rectal tissue [24] and gut [46, 69, 70].

A particular context deserves to be discussed: the diagnosis of HIV infection in babies born to HIV positive mothers. For a long time, the detection of total HIV DNA in PBMCs has been the preferred technique, especially before access to viral load assays [69]. At present, the positive diagnosis of infection remains more difficult, particularly in cases of child infection despite maternal treatment. In such cases, the level of HIV is very low, because the viral replication is relatively blocked by the residual maternal treatment present in the child. So, HIV-DNA level in PBMC could be the only positive marker. In this actual context, there is a need for very sensitive and specific HIV DNA assays [13].

Lastly, total HIV DNA has been a useful marker in many clinical trials, for example, in primary infection [62, 71], in case of treatment with IL2, IL7 or alpha interferon [72–75]. Looking ahead to future clinical interventions aiming at reducing HIV reservoirs, the marker is also suitable, or even indispensable to the first step to select patients and to follow the impact of drugs and combinations [76–79].

The question that arises now is whether this marker could provide information to clinicians for therapeutic management. There are several clinical situations where it can be informative [10]: for example, in patients with long-term efficient treatment, low levels of total HIV-DNA indicate a low risk of disease progression, a low risk of viral rebound and development of drug resistance. On the contrary, in patients with a high level of total HIV-DNA, it is important to explain to them the high risk of viral rebound in case of non-adherence to treatment.

## Conclusions

Among the different markers of HIV persistence, total HIV DNA has a special place because it is by far the most studied, and because the measurement is simple, precise and specific. It can reliably characterize the global size of HIV reservoirs. This marker has already had an undeniable and considerable contribution to reservoir studies, resulting in numerous insights, both in clinical and basic research. This is giving the opportunity to get a large overview of the distribution of HIV reservoir cells in the body, at all stages of HIV disease. Despite its drawback to quantify everything, including defective proviruses, total HIV DNA has enabled major advances, in particular in clinical research. However, there is an urgent need for other standardized markers of HIV reservoirs, in order to complete a panel of accurate tools that can constitute references. The debate should take into account all practical and clinical aspects, and should not sterilize the research, but rather sustain the use of complementary markers, to better explore the mechanisms of viral persistence.
